# The transcriptional regulator *c2h2* accelerates mushroom formation in *Agaricus bisporus*

**DOI:** 10.1007/s00253-016-7574-9

**Published:** 2016-05-21

**Authors:** Jordi F. Pelkmans, Aurin M. Vos, Karin Scholtmeijer, Ed Hendrix, Johan J. P. Baars, Thies Gehrmann, Marcel J. T. Reinders, Luis G. Lugones, Han A. B. Wösten

**Affiliations:** Microbiology, Department of Biology, Utrecht University, Padualaan 8, 3584 CH Utrecht, The Netherlands; Plant Breeding, Wageningen University and Research Centre, 6700 AJ Wageningen, The Netherlands; Delft Bioinformatics Lab, Delft University of Technology, Mekelweg 4, 2628 CD Delft, The Netherlands

**Keywords:** Fungi, Basidiomycete, *Agaricus bisporus*, Mushroom, Transcription factor, Cys2His2

## Abstract

The Cys2His2 zinc finger protein gene *c2h2* of *Schizophyllum commune* is involved in mushroom formation*.* Its inactivation results in a strain that is arrested at the stage of aggregate formation. In this study, the *c2h2* orthologue of *Agaricus bisporus* was over-expressed in this white button mushroom forming basidiomycete using *Agrobacterium*-mediated transformation. Morphology, cap expansion rate, and total number and biomass of mushrooms were not affected by over-expression of *c2h2*. However, yield per day of the *c2h2* over-expression strains peaked 1 day earlier. These data and expression analysis indicate that C2H2 impacts timing of mushroom formation at an early stage of development, making its encoding gene a target for breeding of commercial mushroom strains.

**Electronic supplementary material:**

The online version of this article (doi:10.1007/s00253-016-7574-9) contains supplementary material, which is available to authorized users.

## Introduction

The basidiomycete *Agaricus bisporus* is cultivated globally for the production of white button mushrooms. These fruiting bodies have a relative high protein content and contain fibers, vitamins, minerals, and bioactive compounds. *A. bisporus* is grown on compost formed from wheat straw, horse or chicken manure, and gypsum. During colonization, the compost is topped with a casing layer needed for moisture and microbial flora (Bels-Koning 1950; Flegg 1956; Kalberer 1987). Induction of mushroom formation depends on environmental signals. The volatile 1-octen-3-ol represses early development, while high temperature (i.e., 25 °C instead of 18 °C) inhibits development from smooth to elongated primordia. On the other hand, CO_2_ impacts the number of fruiting bodies that are formed (Noble et al. 2009; Eastwood et al. 2013). Development of *A. bisporus* is a complex process (Kües and Navarro-González 2015). It starts with aggregation of hyphae into hyphal knots (Umar and van Griensven 1997). These structures develop into 1–2-mm initials, also called primordia, that differentiate by forming cap and stem tissues (Umar and van Griensven 1997). Up to 10 % of differentiated primordia develop into mushrooms (Noble et al. 2003). Breaking of the veil of these fruiting bodies enables airborne dispersal of basidiospores that had been formed in the gill tissue within the cap.

Production conditions of white button mushrooms have been optimized with respect to yield and quality of fruiting bodies (Straatsma et al. 2013). However, molecular mechanisms underlying mushroom formation are poorly understood. For instance, transcription factors (TFs) involved in white button development have not been identified so far. Such regulatory proteins have been identified in the model organism *Schizophyllum commune* (Ohm et al. 2010, 2011, 2013). Formation of its fruiting bodies is induced by blue light and is repressed by high CO_2_ (Perkins and Gordon 1969; Niederpruem 1963; Raudaskoski and Viitanen 1982; Ohm et al. 2013). The blue light receptor complex consists of the sensor WC-1 and TF WC-2. Inactivation of *wc-1* and/or *wc-2* results in a blind strain not able to produce aggregates, primordia, and fruiting bodies (Ohm et al. 2013). Strains in which the homeodomain gene *hom2* or the zinc finger TF gene *fst4* have been inactivated are also not able to produce aggregates (Ohm et al. 2010, 2011). In contrast, inactivation of the gene encoding the Cys2His2 zinc finger protein C2H2 results in a strain that does form aggregates but primordia and fruiting bodies are not formed (Ohm et al. 2011). Strains in which genes are inactivated that encode the zinc finger protein Fst3, the GATA type zinc finger protein Gat1, or the homeodomain protein Hom1 form smaller fruiting bodies but in higher numbers (Ohm et al. 2011). These proteins were proposed to play a role in repression of outgrowth of primordia into fruiting bodies or to play a role in expansion of the fruiting body.

Homologs of the *S. commune* TFs involved in fruiting body development have been identified in other mushroom-forming fungi. Expression analysis in *A. bisporus*, *Laccaria bicolor*, and *Coprinopsis cinerea* suggests that mushroom development in the Basidiomycota follows a core regulatory program with species-specific variations that explain differences in morphology and sensitivity to environmental signals (Ohm et al. 2010; Morin et al. 2012; Plaza et al. 2014; Muraguchi et al. 2015). In this study, the *A. bisporus c2h2* homolog was over-expressed in the commercial A15 strain of this mushroom-forming fungus. This resulted in an accelerated rate of mushroom production. Experimental data indicate that C2H2 functions both early and late in mushroom development and that it is an interesting target for breeding of commercial strains.

## Material and methods

### Culture conditions and strains

The heterokaryotic *A. bisporus* strain A15 (obtained from the fungal collection of Plant Breeding Wageningen UR, the Netherlands) and its derivatives AT273-1 and AT273-5 that over-express *c2h2* were routinely grown at 25 °C on malt extract agar medium (MEA; 20 g l^−1^ malt extract agar (BD biosciences, Franklin Lakes, USA), 2.1 g l^−1^ MOPS, pH 7.0). Spawn substrate was produced by heating 75 g of *Sorghum* seeds in water at 100 °C for 20 min, after which 24 g kg^−1^ CaSO_4_ and 6.87 g kg^−1^ CaCO_3_ were added. Spawn was colonized for 3 weeks at 25 °C using two 1-week-old *A. bisporus* colonies as inoculum. Mushrooms were produced by inoculating boxes (40 cm width × 60 cm length × 22 cm height) containing 16 kg phase 2 compost (CNC, Milsbeek, The Netherlands) with 75 g of spawn. Compost temperature was maintained at 25 °C with an air temperature of 22 °C. Relative humidity in growth cells was kept at 95 %, while CO_2_ levels fluctuated between 1500 and 2000 ppm. Ten boxes were inoculated per strain and were randomly distributed in the growth cell. After 16 days, the compost in each box was topped with 7-kg casing layer (CNC, Milsbeek, The Netherlands). Growth was prolonged for 14 days before venting. The casing was manually broken 4 days prior to venting and mixed to create fast regenerative growth and a more equal distribution of *A. bisporus* in the casing layer. Venting resulted in a gradual decrease of compost and air temperature to 19 and 18 °C, respectively. Relative humidity and CO_2_ levels decreased gradually to 85 % and 1200 ppm, respectively. The first buttons were removed from the bed 9 days after venting.

### Analysis of mushroom formation

Photos of casing layer surfaces were taken in a fixed rig at 24-h intervals from venting until the start of the first flush. Emergence of mushrooms and growth rate of the caps were monitored using ImageJ (http://imagej.nih.gov/ij/). Harvesting of mushrooms was done by a professional picker as performed in commercial production. Prior to the flushes, some buttons were removed to open up the space between developing buttons. Fruiting bodies with a diameter between 40 and 60 mm were always harvested, while fruiting bodies with a diameter of ≤40 mm were picked from densely populated areas to provide more space, water, and nutrients to the remaining mushrooms, thereby ensuring optimal yield. Mushrooms were classified as size 40 (mushrooms with a cap ≤40 mm) or size 60 (mushrooms with a cap between 40 and 60 mm). Mushrooms were harvested in two flushes. All mushrooms had reached a size ≥40 mm during the second flush at day 22 and all fruiting bodies were therefore harvested, thus completing the experiment. Yield per box was expressed as the biomass and the number of harvested mushrooms. Height and width of cap and stem were determined of 10 randomly selected mushrooms per box during the peak day of the first flush. Dry weight of the mushrooms was assessed by drying 200 g wet weight fruiting bodies at 100 °C. Relative dry weight is defined as the dry weight compared to the original wet weight.

### Over-expression of c2h2

Primer pair McSpBH_F/McSpBH_R (Table 1) was used to introduce *Pac*I and *Asc*I sites into pBHg (Chen et al. 2000), creating pBHgPA. Gene *c2h2* of *A. bisporus* (ProteinID 230069, http://genome.jgi.doe.gov/Agabi_varbisH97_2) encompassing its coding region with 750-bp up- and downstream sequences was amplified by PCR using genomic DNA of *A. bisporus* A15, primer pair C2h2Abo7wnF/C2h2ABownR (Table 1) and Phusion Hot Start II High-Fidelity DNA polymerase (Thermo Fisher Scientific, Waltham, USA). The amplicon contained *Pac*I and *Asc*I linkers at its 5′ and 3′ ends, respectively, enabling its introduction in pBHgPA that had been cut with *Pac*I and *Asc*I. The resulting plasmid pKS273 was introduced in *Agrobacterium tumefaciens* AGL-1 (Chen et al. 2000). Transformation of *A. bisporus* A15 gills was performed as described (Romaine and Chen 2005). Transformants were screened on MEA plates containing 25 μg ml^−1^ hygromycin, 200 μM cefotaxime, and 100 μg ml^−1^ moxalactum. Transformants were transferred to a second selection plate containing 40 μg ml^−1^ hygromycin, 200 μM cefotaxime, and 17 μg ml^−1^ tetracycline.

**Table 1:** Primers used in this study

Primer name	Sequence
C2h2ABownF	CGCTTAATTAACCTGGCAAAAAAGTGAAC
C2h2ABownR	ATATGGCGCGCCACTACGTCGATGATCATG
McSpBH_F	GATCGTTAATTAAGAATTCAGATCTCAATTGGGCGCGCC
McSpBH_R	GGCGCGCCCAATTGAGATCTGAATTCTTAATTAAC

### Whole genome expression analysis

Mycelium in the casing layer, initials, stage I and stage II buttons, and young fruiting bodies of *A. bisporus* strain A15 were harvested 9 days after venting from two distinct places of the casing bed (thereby creating biological duplos). Due to the method of cultivation at the commercial hand-picking grower Maatschap van den Heuvel, de Rips, The Netherlands, all developmental stages were present on the casing bed at this time point. Casing mycelium was harvested with casing soil. The initials were pooled to obtain sufficient material for RNA isolation. A single stage I button was divided in cap and stipe using a scalpel. A stage II button and a young fruiting body were divided into components of the stipe (skin, underlying tissue and center) and cap (skin, underlying tissue, gill tissue and veil). Samples were immediately frozen in liquid nitrogen. The casing mycelium sample was broken in pieces and kept frozen with liquid nitrogen while harvesting mycelium using cooled tweezers. Samples were homogenized using the TissueLyser II (Qiagen, Düsseldorf, Germany) and RNA was purified using the NucleoSpin RNA kit (Macherey-Nagel, Düren, Germany). Quality was assessed by gel electrophoresis and sent to ServiceXS (Leiden, the Netherlands) for Illumina Next Generation Sequencing. RNA sequencing data have been deposited at NCBI under accession PRJNA309475.

Sequencing revealed between 20,002,387 and 38,840,092 reads. The RNA-Seq pipeline used the TRIMMOMATIC read trimmer version 0.32 (Bolger et al. 2014) to remove low-quality regions and the ILLUMINA adapters from the 125-bp paired end reads. These filtered reads that made up 79–86 % of the initial reads were aligned to the *A. bisporus* v3.0 genome (Sonnenberg, unpublished data) using STAR aligner version 2.4.0f1 (Dobin et al. 2013). The size of the introns was limited to 1500 bp based on the largest intron sizes in the genome annotation provided by the Joint Genome Institute of the Department of Energy (JGI DOE). This resulted in an alignment of 80–93 % of the filtered reads. Abundance estimation was calculated with Cufflinks version 2.1.1 (Trapnell et al. 2012a), and differential expression tests were performed by Cuffdiff using a Benjamini Hochberg false discovery rate of 0.05 (Trapnell et al. 2012b). Proteins annotated to contain a DNA-binding or regulatory protein domain in the InterPro annotation predictions provided by JGI DOE were considered TFs.

### Statistical analyses

Permutations tests were performed to circumvent non-normal distributions of the data. Within each test, 1000.000 permutations were performed. *P* values were corrected with a Benjamini Hochberg procedure using a false discovery rate of 0.05.

## Results

### Whole genome expression analysis

**Fig. 1:**
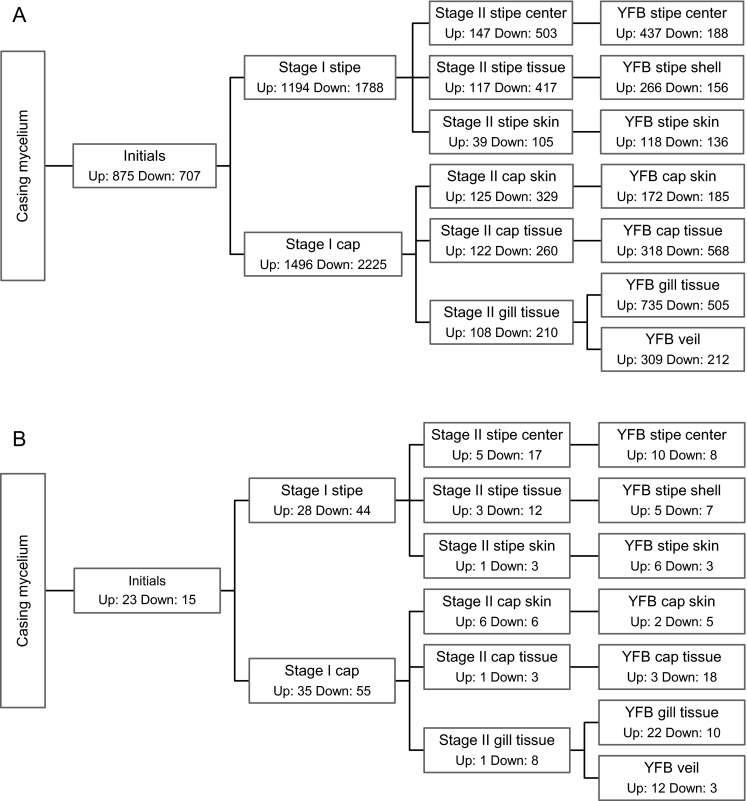
Total up- and downregulated genes (a) and up- and downregulated TF genes (b) comparing initials, stage I buttons, stage II buttons, and young fruiting bodies (YFB) with the preceding developmental stage

RNA composition of casing mycelium, initials, stage I and II buttons, and young fruiting bodies of the commercial *A. bisporus* strain A15 were determined. Initials consisted of 1–2-mm hyphal knots, while stage I buttons were 4–5 mm in diameter. Stage II buttons showed differentiation within the cap and stipe tissue. For instance, gills had developed. Young fruiting bodies were between 15 and 20 mm in diameter and still had their gills covered with veil. Stage I and II buttons and young fruiting bodies were dissected into stipe and cap. The stipes of stage II buttons and young fruiting bodies were subdivided into skin, underlying tissue, and central tissue, while caps were subdivided in skin, underlying tissue, gill tissue, and veil tissue. A total of 875 and 707 genes were up- and downregulated, respectively, in initials when compared to casing mycelium (Fig. 1a). In the stipes and caps of stage I buttons, 1194 and 1496 genes were upregulated when compared to initials, while 1788 and 2225 genes were downregulated. The number of genes that were upregulated in tissues of stage II buttons ranged from 39 to 147, while 105 to 503 genes were downregulated when compared to the caps and stipes of stage I buttons. The number of upregulated genes in young fruiting body tissues ranged from 118 to 735 compared to the stage II button tissues, while 136 to 568 genes were downregulated.

The overall number of TF genes that were upregulated ranged from 1 to 35 when consecutive stages were compared, while the number of downregulated regulatory genes ranged from 3 to 55 (Fig. 1b, Table S1 in the Supplementary Material). The most prominent changes were observed when the initials and caps of stage I buttons were compared (90 differentially expressed TF genes). Only four TF genes were differentially expressed in the transition of the stipes of stage I buttons into the stipe skin of stage II buttons and from stage I caps to stage II cap tissues (Fig. 1b).

Expression of the *A. bisporus* orthologues of the blue light sensor gene *wc-1* and the TF genes *wc-2*, *hom2*, *fst4*, *c2h2*, *fst3*, *gat1*, and *hom1* of *S. commune* (Morin et al. 2012) was analyzed. To this end, expression levels at the different stages of development were compared with mycelium in the casing layer. Transcript levels of *wc-2* and *c2h2* increased >2-fold in initials compared to casing mycelium, while *hom1* levels decreased >2-fold (Table 2; Table S2 in the Supplementary Material). Expression of *wc-1* and *wc-2* was in general higher in aerial structures when compared to the casing mycelium, in stipes when compared to caps, and in outer tissues when compared to inner tissues of the aerial structures. Genes *hom2* and *fst4* were ≥2-fold upregulated when initials had developed in stage I buttons. Like *wc-1* and *wc-2*, they were more highly expressed in stipes when compared to caps, but in this, case there was no difference between outer and inner tissues of the stage II buttons and young fruiting bodies. Gene *c2h2* showed high expression at different stages of fruiting body development. Expression levels ≥4-fold were observed in initials, caps of stage I buttons, gill tissue of stage II buttons, and veil tissue of young fruiting bodies. Expression of *c2h2* was reduced ≥2-fold when compared to casing mycelium in stipe and cap skin and in inner cap tissue of young fruiting bodies. Increased (≥2-fold) levels of *fst3* were only observed in stipes of stage I buttons and in stipe skin and tissue of stage II buttons. Gene *hom1*, and in particular *gat1* was in general downregulated when compared to casing mycelium.

**Table 2:**
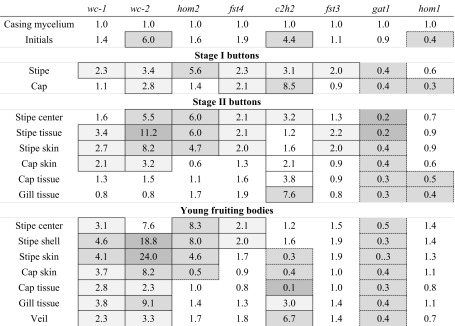
Fold changes in expression during *A. bisporus* development of TF orthologues involved in mushroom formation in *S. commune*

Expression was related to expression in the casing layer*.* Light and dark shading in the dotted line boxes indicate ≥2-fold and ≥4-fold reduced expression in the developmental structures. Light to dark shading in the closed boxes indicates ≥2-fold, ≥4-fold, and ≥10-fold increased expression in the aerial structures

### Over-expression of c2h2 of *A*. *bisporus* results in faster production of mushrooms

Gene *c2h2* (protein ID 230069) of *A. bisporus* shares 79 % identity with its homolog of *S. commune* (protein ID 1194000; http://genome.jgi.doe.gov/Schco3). Expression construct pKS273 (see “Material and methods”) encompassing *A. bisporus* gene *c2h2* was introduced into *A. bisporus* A15 using *A. tumefaciens*-mediated transformation. This resulted in 10 transformants, 2 of which were picked for further analysis. qPCR showed a 30- and a 2.5-fold increase in *c2h2* expression in *A. bisporus* AT273-1 and AT273-5, respectively, when grown on MEA. Growth of these strains on malt extract medium was similar to the parental strain.

**Fig. 2:**
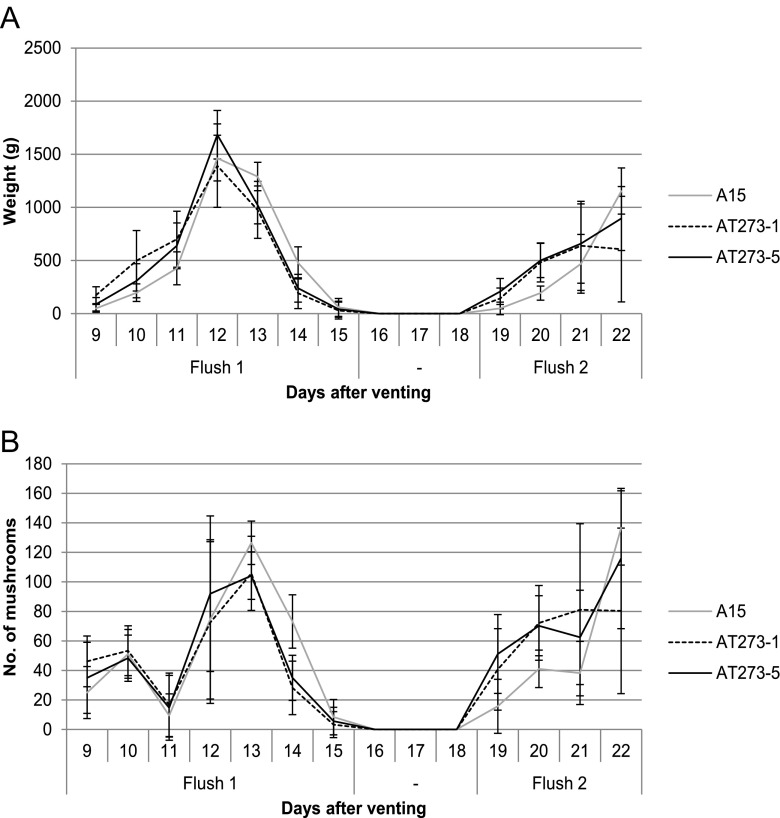
Average biomass (a) and number (b) of A15, AT273-1 and AT273-5 mushrooms per box (n = 10) picked during a 22-day period after venting (t = 0). Bars represent standard deviation

Mushroom production of *A. bisporus* AT273-5 and AT273-1 was assessed in a semi-commercial setting (see “Material and methods”). The first flush started 9 days after venting and progressed until day 14. The second flush took place between day 19 and day 22 (Fig. 2). Biomass of mushrooms harvested at days 9–11 and at days 19–20 was higher for *A. bisporus* AT273-1 when compared to A15 (*p* < 0.01, *p* = 0.01, 0.02, and 0.04 and *p* < 0.01, respectively) (Fig. 2a). *A. bisporus* AT273-5 showed higher harvested mushroom biomass at days 11, 19, and 20 when compared to A15 (*p* = 0.04, and *p* < 0.01 and 0.01, respectively). The latter strain produced more biomass at days 13 and 14 compared to *A. bisporus* AT273-1 (*p* = 0.01 and *p* < 0.01, respectively) and *A. bisporus* AT273-5 (*p* = 0.01 and *p* < 0.01, respectively) and more biomass compared to *A. bisporus* AT273-1 on day 22 (*p* = 0.02). Total production of mushrooms was similar for the three strains (Table 3). A higher number of A15 mushrooms were harvested at day 13 when compared to *A. bisporus* AT273-5 (*p* = 0.01), day 14 compared to both transformants (*p* < 0.01 for both), and day 22 compared to *A. bisporus* AT273-1 (*p* = 0.01 for both). A higher number of *A. bisporus* AT273-1 mushrooms were harvested at days 9 and 20 (*p* = 0.04 and 0.01, respectively) and of *A. bisporus* AT273-5 mushrooms at days 19 and 20 (*p* = 0.01 for both) when compared to A15 (Fig. 2b). Together, these data show that over-expression of *c2h2* accelerates development of mushrooms*.*

**Table 3:** Total weight and number of mushrooms per box of *A. bisporus* strains A15, AT273–1 and AT273–5 after 2 flushes (n = 10)

	Weight (g)		Number of mushrooms	
Strain	Average	Standard deviation	Average	Standard deviation
A15	4197.6	200.1	600.1	114.6
AT273-1	4209.8	439.2	573.1	149.1
AT273-5	4324.4	235.5	634.7	90.9

**Fig. 3:**
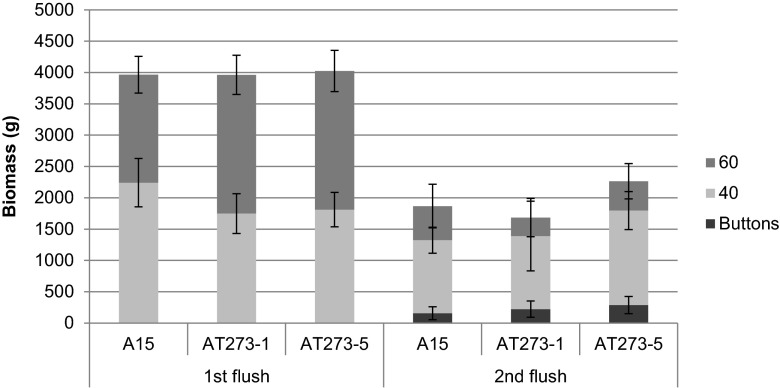
Size composition of A15, AT273-1, and AT273-5 mushrooms harvested during two flushes (n = 100). Bars represent standard deviation

**Fig. 4:**
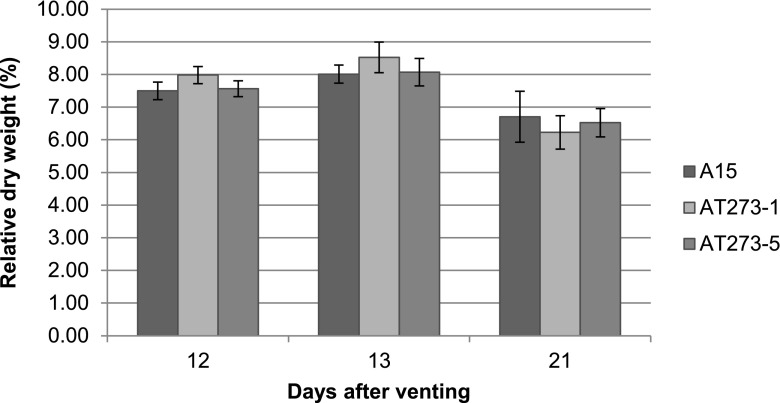
Relative dry weight of mushrooms at days 12, 13, and 21 after vent-off for *A. bisporus* strains A15, AT273-1, and AT273-5 (n = 10). Bars represent standard deviation

Harvested mushrooms were classified based on size 40 (cap ≤40 mm) and size 60 (cap between 40 and 60 mm) (Fig. 3). During the first flush, A15 produced more size 40 mushrooms (57 %) (*p* = 0.01), while the *c2h2* over-expressing strains produced more size 60 mushrooms (56 and 55 %, respectively) (*p* = 0.01 for both). The ratio between cap and stem dimensions were similar for all strains. A relative dry weight of 8 % was found for the mushrooms of the three strains at days 12 and 13 (Fig. 4). All strains produced more size 40 mushrooms in the second flush (64 % for A15 versus 73 and 71 % for AT273-1 and AT273-5) (*p* < 0.01 for all). Relative dry weight at day 21 amounted between 6.2 and 6.7 % for the three strains (Fig. 4). Together, these data show that over-expression of *c2h2* promotes size in the first flush.

**Fig. 5:**
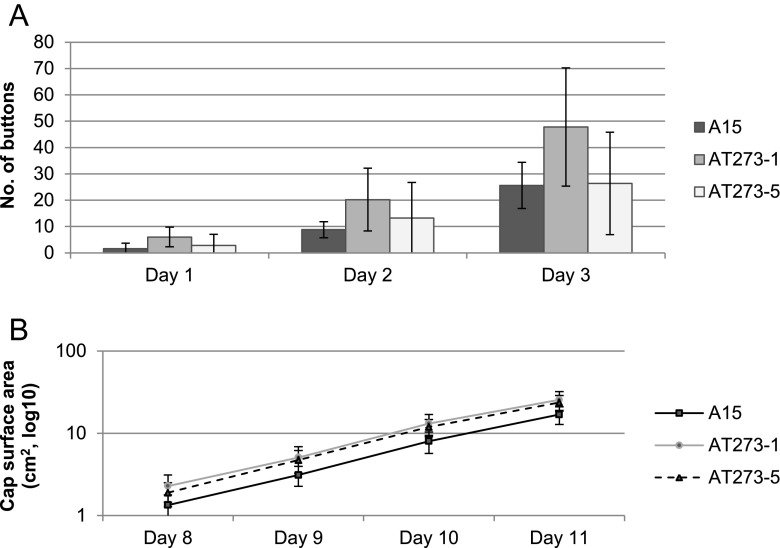
Number of A15, AT273-1, and AT273-5 buttons that had emerged from the casing soil 1, 2, and 3 days after venting (a) and expansion of mushroom caps of the three strains in time (b) (n = 10). Bars represent standard deviation

Mushroom formation was monitored by analyzing photos taken in 24-h intervals. Cap expansion was similar for the three strains (Fig. 5b). The number of buttons emerging from the casing was not significantly different between the three strains but there was a trend that the *c2h2* over-expression strains showed accelerated button emergence (Fig. 5a).

## Discussion

Formation of mushrooms is a highly complex developmental process (Kües 2000). After a submerged mycelium has been formed, hyphae escape the substrate to grow into the air. These hyphae form aggregates with a diameter <1 mm. They result from a single hypha that branches intensely or arise from branches of neighboring aerial hyphae that grow towards and alongside each other. The dark-grown aggregates of *C. cinerea* can develop into different structures depending on light conditions. Continuation of growth in the dark results in formation of sclerotia, while a 12-h day-night cycle induces development of initials. Initials, or primordia, are the first fruiting body-specific structures and can be selectively stained with Janus green (Sánchez and Moore 1999). The switch from aggregate to primordia can be considered key in development since it determines the structure to develop into a sexual reproductive structure. The fact that *c2h2* of *S. commune* is involved in the switch from aggregates to primordia (Ohm et al. 2011) makes it a gene of high interest for fruiting body development. Here, the *c2h2* orthologue of *A. bisporus* was over-expressed resulting in accelerated mushroom formation.

Expression of *c2h2* in different developmental stages was compared to that in casing mycelium. Gene *c2h2* was ≥4-fold upregulated in initials of *A. bisporus*. Upregulation was also found in stage I and stage II buttons, in particular in cap tissue. Expression of *c2h2* was reduced or ≤2-fold upregulated in the tissues of young fruiting bodies with the exception of veil and gill tissues. These expression data indicate that *c2h2* functions early in fruiting body development, while it also seems to have a role in selective tissues of young mushrooms.

Gene *c2h2* of *A. bisporus* was over-expressed in the commercial *A. bisporus* strain A15. Two transformants were selected for phenotypic analysis. These strains, called AT273-1 and AT273-5, displayed 30-fold and 2.5-fold increased *c2h2* expression, respectively. Phenotypes of these strains were similar, indicating that a few fold increased expression of *c2h2* is sufficient to obtain a full effect of over-expression. Morphology, cap expansion rate, and total number and biomass of harvested mushrooms were not affected by over-expression of *c2h2*. However, formation of mushrooms with a cap size ≥4 cm was accelerated. Biomass of harvested mushrooms was increased on days 9 to 11 (first flush) and 19 to 20 (second flush) in *A. bisporus* AT273-1 and days 11, 19, and 20 in *A. bisporus* AT273-5 when compared to A15. On the other hand, A15 produced more biomass on days 13, 14, and 22. The number of harvested mushrooms also indicated accelerated growth of the *c2h2* over-expressors. The fact that expansion rate of mushrooms was similar between the transformants and A15 implies that accelerated mushroom formation is caused at the level of outgrowth of initials. This is supported by a trend that the transformants had formed more initials when compared to A15 1, 2, and 3 days after venting.

It is difficult to compare our results with other whole genome expression studies of mushroom development (Ohm et al. 2010, 2011, 2013; Morin et al. 2012; Plaza et al. 2014; Muraguchi et al. 2015). In this work, RNA from a fertile casing mycelium was used as a reference for differential expression, while Plaza et al. (2014) used fertile vegetative mycelium from complete medium. Ohm et al. (2010, 2011, 2013) compared whole cultures of a sterile monokaryotic vegetative mycelium with whole cultures of the fertile dikaryon at different developmental stages. In contrast, we here used pure developmental structures and tissues. Therefore, we have only focused on expression of genes known to play a role in fruiting body development in *S. commune*. Expression of *c2h2* in *S. commune* is highest in primordia and mature fruiting bodies (Ohm et al. 2011), which is in agreement with the findings in *A. bisporus*. The genes encoding the blue light sensing complex components Wc-1 and Wc-2 are also most highly expressed in primordia and fruiting bodies of *S. commune.* This agrees with the finding that the *A. bisporus* homologs were more highly expressed in aerial structures when compared to casing mycelium, in stipe tissue when compared to cap tissue, and in outer tissues of the aerial structures when compared to inner tissues. *A. bisporus* does not require blue light to produce mushrooms. Yet, blue light sensing is also required to induce UV light-related DNA damage repair (e.g., photolyase) and in conversion of toxic porphyrin intermediates in heme (ferrochelatase) (Ohm et al. 2013). Expression of *hom2* of *S. commune* does not change during development until the stage of mature fruiting bodies when expression drops. Expression of *fst3* and *fst4* is considered constitutive in *S. commune*. Gene *hom2* was ≥2-fold over-expressed when initials had developed into stage I buttons. Like *wc-1* and *wc-2*, expression of *hom2*, *fst4*, and *fst3* were more highly expressed in stipes when compared to caps but only small differences were observed between outer and inner tissues of the stage II buttons and young fruiting bodies. Genes *gat1* and *hom1* of *S. commune* are most highly expressed in late stages of mushroom development, although their upregulation is modest. These genes have a different expression profile in *A. bisporus.* They were generally downregulated in the developmental structures when compared to casing mycelium. This effect was most prominent for *gat1.* It may thus be that their role in *S. commune* and *A. bisporus* is different. Recently, expression profiles of *wc-2*, *hom2*, *fst4*, *c2h2*, *fst3*, *hom1* and *gat1* were determined in stipe and cap during fruiting body development in *C. cinerea* (Muraguchi et al. 2015). Expression of *wc-2* increased during initial development and was higher in the stipe when compared to cap. Genes *fst4* and *fst3* were also more highly expressed in the stipe. This is similar to the *A. bisporus* expression profiles presented in this study. Expression of *hom2* remained constant during the early development, but in contrast to *A. bisporus*, was higher in the cap during later stages. Transcript levels of *gat1* slightly increased during development in *C. cinerea*, while they diminished in *A. bisporus* at this stage. Expression of *c2h2* was higher in the cap compared to the stipe tissues early in development while this was reversed later in development, a situation similar to *A. bisporus*. Together, these data support the view that mushroom development in the Basidiomycota follows a core regulatory program with species-specific variations that may explain differences in morphology and sensitivity to environmental signals.

## Electronic Supplementary Material

ESM 1 (PDF 230 kb)
