# Supplementary material for: The transcriptional regulator c2h2 accelerates mushroom formation in Agaricus bisporus
Source: Appl Microbiol Biotechnol. 2016 May 21;100:7151–9. doi: 10.1007/s00253-016-7574-9 (PMC4947489; doi:10.1007/s00253-016-7574-9)
Supplement: Supplementary file 1 — (PDF 230 kb) [file 253_2016_7574_MOESM1_ESM.pdf]

Applied Microbiology and Biotechnology

The transcriptional regulator *c2h2* accelerates mushroom formation in *Agaricus bisporus*

Jordi F. Pelkmans[a], Aurin M. Vos[a], Karin Scholtmeijer[a,b], Ed Hendrix[b], Johan J.P. Baars[b], Thies Gehrmann[c], Marcel J. T. Reinders[c], Luis G. Lugones[a], Han A.B. Wösten[a]

[a]Microbiology, Utrecht University, Padualaan 8, 3584 CH Utrecht, The Netherlands.

[b]Plant Breeding, Wageningen University and Research Centre, 6700 AJ Wageningen, The Netherlands

[c]Delft Bioinformatics Lab, Delft University of Technology, Mekelweg 4, 2628 CD Delft, The Netherlands.

Corresponding author

Prof. dr. Han A.B. Wösten

Department of Microbiology, Utrecht University

Padualaan 8, 3584 CH Utrecht

The Netherlands

Telephone: 31 30 2533448

Fax: 31 30 2513655

E-mail: h.a.b.wosten@uu.nl

Supplemental Table S1 ProteinIDs of transcription factor genes differentially expressed in initials, stage I buttons, stage II buttons, and young fruiting bodies (YFB) when compared to the preceding developmental stage.

Initials

**Upregulated**			**Downregulated**		
**ProteinID**	**Annotation**	**Fold-change**	**ProteinID**	**Annotation**	**Fold-change**
185241	Fungal transcriptional regulatory protein, N-terminal	1.9	141117	HMG-I and HMG-Y, DNA-binding	0.5
56847	High mobility group box, HMG	INF	71198	Fungal specific transcription factor	0.4
199498	TEA/ATTS	5.6	204681	Fungal transcriptional regulatory protein, N-terminal	0.3
224104	Homeobox	1.9	229742	Zinc finger, C2H2-type	0.5
114694	Zinc finger, C2H2-type	2.0	206880	Zinc finger, C2H2-type	0.4
117682	Bacterial regulatory protein, MarR	2.5	189981	Zinc finger, C2H2-type	0.4
118034	Homeodomain-like	1.9	179892	High mobility group box, HMG	0.3
116879	Zinc finger, C2H2-type	2.5	180441	Basic helix-loop-helix dimerisation region bHLH	0.5
116860	Homeodomain-like SANT, DNA-binding	1.8	189288	Homeobox	0.5
132586	Basic-leucine zipper (bZIP)	3.8	192433	Homeobox	0.4
199557	Fungal specific transcription factor	2.1	**137362**	Centromere protein B, helix-turn-helix Homeodomain-like	0.2
75421	SANT, glycoside hydrolase	2.5	122840	Zinc finger, C2H2-type	0.4
185465	HMG-I and HMG-Y, DNA-binding	1.8	61584	Basic helix-loop-helix dimerisation region bHLH	0.4
223670	Fungal specific transcription factor	1.9			
192334	Zinc finger, C2H2-type	1.9			
117665	Zinc finger, C2H2-type	2.2			
230069	Zinc finger, C2H2-type	4.4			
120238	High mobility group box, HMG	4.2			
190401	Zinc finger, GATA-type	6.0			

Stage I buttons stipe

**Upregulated**			**Downregulated**		
**ProteinID**	**Annotation**	**Fold-change**	**ProteinID**	**Annotation**	**Fold-change**
141117	HMG-I and HMG-Y, DNA-binding	3.0	113594	Fungal specific transcription factor	0.5
56560	High mobility group box, HMG	2.1	74326	Zinc finger, NF-X1-type	0.5
196071	Basic leucine zipper	2.0	56847	High mobility group box, HMG	INF
147784	SANT, DNA-binding	2.3	204101	Zinc finger, C2H2-type	INF
147361	Homeobox	2.1	199498	TEA/ATTS	0.5
196103	Zinc finger, C2H2-type	2.0	116501	Basic helix-loop-helix dimerisation region bHLH	0.4
**117837**	HMG-I and HMG-Y, DNA-binding	1.8	68386	Homeodomain-like SANT, DNA-binding	0.6
**223664**	Fungal specific transcription factor	2.0	78651	Zinc finger, Rad18-type putative	0.4
**208468**	High mobility group box, HMG	2.2	194792	Basic-leucine zipper (bZIP) transcription factor	0.5
78141	Zinc finger, C2H2-type	2.0	123182	Fungal specific transcription factor	0.4
**197611**	Homeodomain-like	1.9	117682	Bacterial regulatory protein, MarR	0.5
**215897**	Zinc finger, DHHC-type	2.0	187161	Basic-leucine zipper (bZIP) transcription factor	0.6
138604	Fungal transcriptional regulatory protein, N-terminal	4.1	121841	Zinc finger, GATA-type	0.4
191883	SANT, glycoside hydrolase	2.0	132586	Basic leucine zipper	0.4
179892	High mobility group box, HMG	2.3	199557	Fungal specific transcription factor	0.3
187697	Fungal specific transcription factor	1.8	**229713**	Homeodomain-like	0.6
136982	Zinc finger, GATA-type	2.6	227329	Zinc finger, TFIIS-type	0.3
189288	Homeobox	2.2	211033	Lambda repressor-like, DNA-binding	0.1
119147	Zinc finger, GATA-type	2.6	192389	p53-like transcription factor, DNA-binding	0.3

**212592**		1.1	75421	SANT, glycoside hydrolase	0.6
61584	Basic helix-loop-helix dimerisation region bHLH	2.3	183343	Fungal transcriptional regulatory protein, N-terminal	0.4
191328	Fungal specific transcription factor	1.9	190699	Zinc finger, C2H2-type	0.6
192725	Homeobox	3.4	**218115**	DnaJ	0.5
197030	High mobility group box, HMG	2.9	222360	Fungal specific transcription factor	0.6
			187173	Basic helix-loop-helix dimerisation region bHLH	0.5
			116656		0.2
			123539		0.4
			122941	Zinc finger, C2H2-type	0.2
			189365	Zinc finger, C2H2-type	0.4
			192334	Zinc finger, C2H2-type	0.5
			195724	Zinc finger, GATA-type	0.5
			115873	Zinc finger, C2H2-type	0.3
			191564	Homeodomain-like SANT, DNA-binding	0.5
			193132	Zinc finger, C2H2-type	0.4
			193980	Zinc finger, C2H2-type	0.5
			194929		0.5
			177769	Zinc finger, C2H2-type	0.5
			188638	High mobility group box, HMG	0.3
			191136	Fungal transcriptional regulatory protein, N-terminal	0.5

Stage I buttons cap

Upregulated			Downregulated		
**ProteinID**	**Annotation**	**Fold-change**	**ProteinID**	**Annotation**	**Fold-change**
141117	HMG-I and HMG-Y, DNA-binding	2.7	113594	Fungal specific transcription factor	0.2
56560	High mobility group box, HMG	2.5	74326	Zinc finger, NF-X1-type	0.4
199498	TEA/ATTS	2.0	56847	High mobility group box, HMG	INF
196071	Basic leucine zipper	2.3	116501	Basic helix-loop-helix dimerisation region bHLH	0.4
147784	SANT, DNA-binding	2.8	68386	Homeodomain-like SANT, DNA-binding	0.6
147361	Homeobox	2.4	78651	Zinc finger, Rad18-type putative	0.5
196103	Zinc finger, C2H2-type	1.8	**191917**	Basic-leucine zipper (bZIP) transcription factor	0.5
**193519**	SANT, DNA-binding	2.1	**206331**	Fungal specific transcription factor	0.4
**225055**	Homeodomain-like SANT, DNA-binding	1.9	**73014**	Fungal transcriptional regulatory protein, N-terminal	0.5
**135160**	Transcription factor CBF/NF-Y/archaeal histone	2.0	194792	Basic-leucine zipper (bZIP) transcription factor	0.3
78141	Zinc finger, C2H2-type	1.9	**182409**	Basic-leucine zipper (bZIP) transcription factor	0.4
79223	Zinc finger, C2H2-type	2.8	123182	Fungal specific transcription factor	0.5
**70431**	ssDNA-binding transcriptional regulator	2.2	117682	Bacterial regulatory protein, MarR	0.4
**221859**	Basic-leucine zipper (bZIP) transcription factor	1.9	**116508**	Zinc finger, C2H2-type	0.2
**219430**	DNA-binding, Ankyrin	2.1	187161	Basic-leucine zipper (bZIP) transcription factor	0.5
138604	Fungal transcriptional regulatory protein, N-terminal	2.1	121841	Zinc finger, GATA-type	0.3
191883	SANT, glycoside hydrolase	2.1	132586	Basic leucine zipper	0.4
**71368**	DNA-binding, Ankyrin	1.9	199557	Fungal specific transcription factor	0.2
179892	High mobility group box, HMG	3.1	**219861**	Fungal specific transcription factor	0.4
189288	Homeobox	3.1	227329	Zinc finger, TFIIS-type	0.2
117665	Zinc finger, C2H2-type	4.5	211033	Lambda repressor-like, DNA-binding	INF
**191725**	Zinc finger, CCHC-type	2.0	192389	p53-like transcription factor, DNA-binding	0.4
191645	HMG High mobility group box, HMG1/HMG2	2.6	75421	SANT, glycoside hydrolase	0.6

230069	Zinc finger, C2H2-type	2.0	138736	Fungal transcriptional regulatory protein, N-terminal	0.2
**190999**	High mobility group box, HMG	2.1	183343	Fungal transcriptional regulatory protein, N-terminal	0.4
193208	Zinc finger, C2H2-type	3.3	222360	Fungal specific transcription factor	0.6
**175561**	Homeodomain-like SANT, DNA-binding	1.8	187173	Basic helix-loop-helix dimerisation region bHLH	0.3
**134080**	MBF	2.0	182895	Basic helix-loop-helix dimerisation region bHLH	0.3
**190684**	Homeodomain-like SANT, DNA-binding	2.2	116656		0.1
191328	Fungal specific transcription factor	2.1	122941	Zinc finger, C2H2-type	0.1
197030	High mobility group box, HMG	3.7	189365	Zinc finger, C2H2-type	0.3
			192334	Zinc finger, C2H2-type	0.5
			**192345**	Basic leucine zipper	0.5
			148501	Zinc finger, C2H2-type	0.5
			122836	Zinc finger, GATA-type	0.2
			195724	Zinc finger, GATA-type	0.5
			115873	Zinc finger, C2H2-type	0.2
			115875	Zinc finger, C2H2-type	0.5
			191490	Zinc finger, C2H2-type	0.3
			191564	Homeodomain-like SANT, DNA-binding	0.1
			193132	Zinc finger, C2H2-type	0.4
			193980	Zinc finger, C2H2-type	0.3
			194007	Fungal transcriptional regulatory protein, N-terminal	0.5
			194929		0.4
			122840	Zinc finger, C2H2-type	0.5
			177769	Zinc finger, C2H2-type	0.4
			**183616**	Fungal transcriptional regulatory protein, N-terminal	0.5
			184201	Zinc finger, C2H2-type	0.2
			188638	High mobility group box, HMG	0.2
			190401	Zinc finger, GATA-type	0.5
			191136	Fungal transcriptional regulatory protein, N-terminal	0.4

Stage II stipe center

Upregulated			Downregulated		
**ProteinID**	**Annotation**	**Fold-change**	**ProteinID**	**Annotation**	**Fold-change**
**208769**	Zinc finger, PARP-type	2.9	113594	Fungal specific transcription factor	0.3
**117743**	Fungal specific transcription factor	3.3	224104	Homeobox	0.1
204206	Fungal transcriptional regulatory protein, N-terminal Zinc finger, C2H2-type	5.3	199557	Fungal specific transcription factor	0.5
224564	Fungal transcriptional regulatory protein, N-terminal Zinc finger, C2H2-type	4.6	178849	Fungal transcriptional regulatory protein, N-terminal	0.5
138736	Fungal transcriptional regulatory protein, N-terminal	3.9	223295	Fungal specific transcription factor	0.5
			122941	Zinc finger, C2H2-type	0.3
			189365	Zinc finger, C2H2-type	0.5
			117665	Zinc finger, C2H2-type	0.1
			195724	Zinc finger, GATA-type	0.4
			115873	Zinc finger, C2H2-type	0.4
			191490	Zinc finger, C2H2-type	0.1
			191564	Homeodomain-like SANT, DNA-binding	0.4
			193208	Zinc finger, C2H2-type	0.1
			184201	Zinc finger, C2H2-type	0.2
			116512		1.8

Stage II stipe tissue

Upregulated			Downregulated		

ProteinID	Annotation	Fold-change	ProteinID	Annotation	Fold-change
204101	Zinc finger, C2H2-type	1.3	185241	Fungal transcriptional regulatory protein, N-terminal	0.5
177333	Zinc finger, GATA-type	1.7	113594	Fungal specific transcription factor	0.3
190401	Zinc finger, GATA-type	3.3	223451	Zinc finger, C2H2-type	0.4
			224104	Homeobox	0.3
			224564	Fungal transcriptional regulatory protein, N-terminal Zinc finger, C2H2-type	0.5
			192389	p53-like transcription factor, DNA-binding	0.3
			117665	Zinc finger, C2H2-type	0.2
			230069	Zinc finger, C2H2-type	0.4
			191490	Zinc finger, C2H2-type	0.2
			193208	Zinc finger, C2H2-type	0.2
			197030	High mobility group box, HMG	0.5

Stage II stipe skin

Upregulated			Downregulated		
ProteinID	Annotation	Fold-change	ProteinID	Annotation	Fold-change
190401	Zinc finger, GATA-type	2.4	224564	Fungal transcriptional regulatory protein, N-terminal Zinc finger, C2H2-type	0.5
			138736	Fungal transcriptional regulatory protein, N-terminal	INF
			230069	Zinc finger, C2H2-type	0.5

Stage II cap skin

Upregulated			Downregulated		
ProteinID	Annotation	Fold-change	ProteinID	Annotation	Fold-change
185241	Fungal transcriptional regulatory protein, N-terminal	2.2	122355	Homeobox	0.6
132586	Basic leucine zipper	2.2	138736	Fungal transcriptional regulatory protein, N-terminal	INF
178849	Fungal transcriptional regulatory protein, N-terminal	2.4	230069	Zinc finger, C2H2-type	0.2
180216	Transcription factor, STE-like	1.8	115875	Zinc finger, C2H2-type	0.5
192433	Homeobox	1.8	192725	Homeobox	0.5
194007	Fungal transcriptional regulatory protein, N-terminal	1.9	116512		0.9

Stage II cap tissue

Upregulated			Downregulated		
ProteinID	Annotation	Fold-change	ProteinID	Annotation	Fold-change
194007	Fungal transcriptional regulatory protein, N-terminal	1.8	79223	Zinc finger, C2H2-type	0.5
			230069	Zinc finger, C2H2-type	0.4
			116512		0.8

Stage II gill tissue

Upregulated			Downregulated		
ProteinID	Annotation	Fold-change	ProteinID	Annotation	Fold-change
138736	Fungal transcriptional regulatory protein, N-terminal	3.1	185241	Fungal transcriptional regulatory protein, N-terminal	0.5
			199498	TEA/ATTS	0.4
			79223	Zinc finger, C2H2-type	0.4
			192389	p53-like transcription factor, DNA-binding	0.5
			189365	Zinc finger, C2H2-type	0.6
			191490	Zinc finger, C2H2-type	0.4
			193208	Zinc finger, C2H2-type	0.5

			190401	Zinc finger, GATA-type	0.3

Young fruiting bodies stipe center

Upregulated			Downregulated		
**ProteinID**	**Annotation**	**Fold-change**	**ProteinID**	**Annotation**	**Fold-change**
185241	Fungal transcriptional regulatory protein, N-terminal	2.3	223451	Zinc finger, C2H2-type	0.3
113594	Fungal specific transcription factor	2.5	147784	SANT, DNA-binding	0.6
116501	Basic helix-loop-helix dimerisation region bHLH	2.4	138604	Fungal transcriptional regulatory protein, N-terminal	0.5
121272	Fungal specific transcription factor	3.1	138736	Fungal transcriptional regulatory protein, N-terminal	0.4
199557	Fungal specific transcription factor	2.6	117665	Zinc finger, C2H2-type	0.3
180441	Basic helix-loop-helix dimerisation region bHLH	1.7	230069	Zinc finger, C2H2-type	0.4
192433	Homeobox	2.0	197030	High mobility group box, HMG	0.3
195724	Zinc finger, GATA-type	2.7			
191490	Zinc finger, C2H2-type	4.1			
191564	Homeodomain-like SANT, DNA-binding	3.9			

Young fruiting bodies stipe shell

Upregulated			Downregulated		
**ProteinID**	**Annotation**	**Fold-change**	**ProteinID**	**Annotation**	**Fold-change**
116501	Basic helix-loop-helix dimerisation region bHLH	2.3	224104	Homeobox	0.4
121272	Fungal specific transcription factor	2.6	138604	Fungal transcriptional regulatory protein, N-terminal	0.5
204206	Fungal transcriptional regulatory protein, N-terminal Zinc finger, C2H2-type	5.1	185465	HMG-I and HMG-Y, DNA-binding	0.6
224564	Fungal transcriptional regulatory protein, N-terminal Zinc finger, C2H2-type	3.4	178849	Fungal transcriptional regulatory protein, N-terminal	0.4
180441	Basic helix-loop-helix dimerisation region bHLH	1.7	117665	Zinc finger, C2H2-type	0.2
			193208	Zinc finger, C2H2-type	0.2

Young fruiting bodies stipe skin

Upregulated			Downregulated		
**ProteinID**	**Annotation**	**Fold-change**	**ProteinID**	**Annotation**	**Fold-change**
189243	Zinc finger, C2H2-type	4.2	178849	Fungal transcriptional regulatory protein, N-terminal	0.4
116501	Basic helix-loop-helix dimerisation region bHLH	2.2	117665	Zinc finger, C2H2-type	0.5
204206	Fungal transcriptional regulatory protein, N-terminal Zinc finger, C2H2-type	1.8	230069	Zinc finger, C2H2-type	0.2
180441	Basic helix-loop-helix dimerisation region bHLH	1.7			
190401	Zinc finger, GATA-type	2.9			

Young fruiting bodies cap skin

Upregulated			Downregulated		
**ProteinID**	**Annotation**	**Fold-change**	**ProteinID**	**Annotation**	**Fold-change**
189243	Zinc finger, C2H2-type	2.4	132586	Basic leucine zipper	0.5
190401	Zinc finger, GATA-type	2.5	178849	Fungal transcriptional regulatory protein, N-terminal	0.3
			180216	Transcription factor, STE-like	0.5
			191645	HMG High mobility group box, HMG1/HMG2	0.5
			230069	Zinc finger, C2H2-type	0.2

Young fruiting bodies cap tissue

Upregulated			Downregulated		
**ProteinID**	**Annotation**	**Fold-change**	**ProteinID**	**Annotation**	**Fold-change**
189243	Zinc finger, C2H2-type	2.3	74659	Zinc finger, PARP-type	0.3
**192596**	Fungal transcriptional regulatory protein, N-terminal	2.0	199498	TEA/ATTS	0.4
122836	Zinc finger, GATA-type	2.3	78651	Zinc finger, Rad18-type putative	0.5
			224104	Homeobox	0.2
			192389	p53-like transcription factor, DNA-binding	0.3
			185465	HMG-I and HMG-Y, DNA-binding	0.5
			223670	Fungal specific transcription factor	0.5
			178849	Fungal transcriptional regulatory protein, N-terminal	0.4
			223295	Fungal specific transcription factor	0.5
			190080	Zinc finger, TFIIS-type	0.4
			117665	Zinc finger, C2H2-type	0.2
			191645	HMG High mobility group box, HMG1/HMG2	0.4
			230069	Zinc finger, C2H2-type	INF
			191490	Zinc finger, C2H2-type	0.2
			116512		0.8

Young fruiting bodies gill tissue

Upregulated			Downregulated		
**ProteinID**	**Annotation**	**Fold-change**	**ProteinID**	**Annotation**	**Fold-change**
185241	Fungal transcriptional regulatory protein, N-terminal	4.4	122355	Homeobox	0.5
189243	Zinc finger, C2H2-type	3.4	223451	Zinc finger, C2H2-type	0.4
199498	TEA/ATTS	3.6	196103	Zinc finger, C2H2-type	0.5
116501	Basic helix-loop-helix dimerisation region bHLH	5.8	224564	Fungal transcriptional regulatory protein, N-terminal Zinc finger, C2H2-type	0.5
224104	Homeobox	3.0	138736	Fungal transcriptional regulatory protein, N-terminal	INF
222207	TEA/ATTS	2.7	189288	Homeobox	0.5
132586	Basic leucine zipper	3.0	190080	Zinc finger, TFIIS-type	0.4
79223	Zinc finger, C2H2-type	5.9	191645	HMG High mobility group box, HMG1/HMG2	0.4
192389	p53-like transcription factor, DNA-binding	2.5	230069	Zinc finger, C2H2-type	0.4
182895	Basic helix-loop-helix dimerisation region bHLH	2.3			
223295	Fungal specific transcription factor	2.0			
189365	Zinc finger, C2H2-type	2.7			
192433	Homeobox	2.8			
148501	Zinc finger, C2H2-type	2.4			
115873	Zinc finger, C2H2-type	2.5			
191490	Zinc finger, C2H2-type	2.0			
193208	Zinc finger, C2H2-type	2.7			
194929		1.7			
188638	High mobility group box, HMG	3.4			
190401	Zinc finger, GATA-type	11.4			
182786	Basic-leucine zipper (bZIP) transcription factor	1.8			

Young fruiting bodies veil

Upregulated			Downregulated		
**ProteinID**	**Annotation**	**Fold-change**	**ProteinID**	**Annotation**	**Fold-change**
185241	Fungal transcriptional regulatory protein, N-terminal	2.1	223451	Zinc finger, C2H2-type	0.5

189243	Zinc finger, C2H2-type	2.9	190080	Zinc finger, TFIIS-type	0.4
224104	Homeobox	2.3			
222207	TEA/ATTS	1.9			
79223	Zinc finger, C2H2-type	3.0			
182895	Basic helix-loop-helix dimerisation region bHLH	1.8			
189365	Zinc finger, C2H2-type	2.1			
117665	Zinc finger, C2H2-type	1.9			
193208	Zinc finger, C2H2-type	2.4			
188638	High mobility group box, HMG	2.2			
190401	Zinc finger, GATA-type	4.2			

Analysis has been performed in version 3 of the *A. bisporus* genome. Since this improved version is not yet published we use the ProteinIDs and corresponding annotations of version 2 of the genome sequence (v2, http://genome.jgi.doe.gov/Agabi_varbisH97_2). In version 3 some genes, highlighted with green shading, have been newly identified as transcription factor genes. ProteinIDs in bold and orange shading are exclusively differentially expressed at the particular stage/tissue. Some genes have been annotated as transcription factors in version 3 that did not exist in version 2. Since these genes do not have a proteinID in version 2, they are not shown in this table.

Table S2. Expression levels (FPKM) of orthologues of regulators of mushroom formation in *S. commune* during development of *A. bisporus.*

	wc-1	*wc-2*	*hom2*	*fst4*	*c2h2*	*fst3*	*gat1*	*hom1*
Casing Myc	64	139	50	120	45	74	284	438
Initials	88	827	82	233	196	84	250	166
**Stage I buttons**								
Stipe	145	474	281	289	140	155	113	250
Cap	71	382	69	249	384	65	119	150
**Stage II buttons**								
Stipe Center	104	772	301	251	143	93	50	312
Stipe Tissue	214	1558	302	251	55	166	69	378
Stipe Skin	174	1145	234	242	70	152	118	386
Cap Skin	137	448	32	155	94	63	123	276
Cap Tissue	86	212	55	192	172	69	95	211
Gill Tissue	51	111	83	234	343	61	86	179
**Young fruiting bodies**								
Stipe Center	197	1050	416	286	52	113	135	618
Stipe Shell	297	2620	405	246	73	138	96	627
Stipe Skin	264	3335	232	198	13	139	77	585
Cap Skin	236	1136	25	105	18	75	105	472
Cap Tissue	181	328	51	99	6	75	72	351
Gill Tissue	242	1267	69	161	137	101	116	492
Veil	149	462	86	221	302	98	115	296
